# Analysis of the fetal cardio-electrohysterographic coupling at the third trimester of gestation in healthy women by Bivariate Phase-Rectified Signal Averaging

**DOI:** 10.1371/journal.pone.0236123

**Published:** 2020-07-10

**Authors:** José Eduardo Montero-Nava, Adriana Cristina Pliego-Carrillo, Claudia Ivette Ledesma-Ramírez, Miguel Ángel Peña-Castillo, Juan Carlos Echeverría, Gustavo Pacheco-López, José Javier Reyes-Lagos

**Affiliations:** 1 Autonomous University of The State of Mexico (UAEMex), Faculty of Medicine, Toluca, Mexico; 2 Metropolitan Autonomous University (UAM), Campus Iztapalapa, Basic Sciences and Engineering Division, Mexico City, Mexico; 3 Metropolitan Autonomous University (UAM), Campus Lerma, Biological and Health Sciences Division, Lerma, Mexico; University of Mississippi Medical Center, UNITED STATES

## Abstract

**Introduction:**

The fetal cardio-electrohysterographic coupling (FCEC) is defined as the influence of the uterine electrical activity on fetal heart rate. FCEC has been mainly evaluated by visual analysis of cardiotocographic data during labor; however, this physiological phenomenon is poorly explored during the antenatal period. Here we propose an approach known as Bivariate Phase-Rectified Signal Averaging analysis (BPRSA) to assess such FCEC in the late third trimester of low-risk pregnancies. We hypothesized that BPRSA is a more reliable measure of FCEC than visual analysis and conventional measures such as cross-correlation, coherence, and cross-sample entropy. Additionally, by using BPRSA it is possible to detect FCEC even from the third trimester of pregnancy.

**Material and methods:**

Healthy pregnant women in the last third trimester of pregnancy (36.6 ± 1.8 gestational weeks) without any clinical manifestation of labor were enrolled in the Maternal and Childhood Research Center (CIMIGen), Mexico City (n = 37). Ten minutes of maternal electrohysterogram (EHG) and fetal heart rate (FHR) data were collected by a transabdominal non-invasive device. The FCEC was quantified by the coefficient of coherence, the maximum normalized cross-correlation, and the cross-sample entropy obtained either from the EHG and FHR raw signals or from the corresponding BPRSA graphs.

**Results:**

We found that by using BPRSA, the FCEC was detected in 92% cases (34/37) compared to 48% cases (18/37) using the coefficient of coherence between the EHG and FHR raw signals. Also, BPRSA indicated FCEC in 82% cases (30/37) compared to 30% cases (11/37) using the maximum normalized cross-correlation. By comparing the analyses, the BPRSA evidenced higher FCEC in comparison to the coupling estimated from the raw EHG and FHR signals.

**Conclusions:**

Our results support the consideration that in the third trimester of pregnancy, the fetal heart rate is also influenced by uterine activity despite the emerging manifestation of this activity before labor. To quantify FCEC, the BPRSA can be applied to FHR and EHG transabdominal signals acquired in the third trimester of pregnancy.

## 1. Introduction

The third trimester of pregnancy is characterized by a gradual increase in the excitability of the uterine muscle; it is mediated by increasing concentrations of prostaglandins, promoted by estrogen [1]. Estrogen also increases the expression of oxytocin receptors and myometrial gap junctions, facilitating the ability of the uterus to show coordinated contractions [1]. In the third trimester of pregnancy, uterine contractions increase progressively achieving frequencies of one to two contractions in 20 minutes [2]. These contractions are often referred to as pre-labor practice contractions [3]. In coincidence with higher uterine activity, the average amplitude of the uterine electromyogram or electrohysterogram (EHG) increases as a function of the progression of pregnancy [4].

Interestingly, previous studies have shown that the uterine contractions are also associated with the acceleration of the fetal heart rate in both healthy and growth-restricted fetuses [5,6]. Some studies indicate that the intensity of the antenatal uterine activity has an increased progressive effect on FHR in the second and third trimester of pregnancy, which may represent a physiological challenge for the developing fetal cardiovascular system [6]. In sum, antenatal contractions might be better conceptualized as: mother-labor-practice / fetus-neurodevelopmental-early stimulation.

Fetal heart rate (FHR) monitoring by transabdominal recordings can be used to provide noninvasive autonomic markers of fetal wellbeing [7,8]. Changes of FHR depict the influence of the maturation of the fetal autonomic nervous system during pregnancy progression [9]. Signal processing techniques can also provide a reproducible numerical assessment of the relationship between FHR and EHG [10,11]. This physiological phenomenon is known as the fetal cardio-electrohysterographic coupling (FCEC), and it indicates the influence of uterine electrical activity on FHR. At present, FCEC is indirectly evaluated by clinicians using visual analysis of cardiotocographic data during labor [10], which is subjective and demands specialized individual training. Thus, such phenomenon has been poorly explored during the antenatal period. Only few studies have concluded that the uterine contractions lead to transient fetal heart rate accelerations during the second and third trimester of pregnancy [12].

The FCEC is a novel potential biomarker that may provide additional information about fetal neurodevelopment from the perspective of coupling analysis [11]. Different computational methods have been used to determine the FCEC during labor. In particular, Casati et al. applied a method known as Bivariate Phase-Rectified Signal Averaging analysis (BPRSA) to explore the FCEC in parturient women. They found a significant coupling in 90.3% of the cases analyzed by BPRSA in comparison to an underestimated 24.2% of cases analyzed by the raw coherence between the FHR and EHG signals [10]. This method overcomes the limitations of non-stationary and background noise that are typical of the FHR signals. However, it is unknown if that physiological coupling mechanism is also manifested earlier in the third trimester of pregnancy. It is also relevant to determine if other techniques, such as the cross-sample entropy and cross-correlation used to evaluate couplings in other physiological contexts [13,14], can detect significant FCEC couplings between EHG and FHR as well.

This study aimed to compare the BPRSA with other analyses to determine its reliability as a tool for quantifying the FCEC in healthy pregnant women in the antenatal period. Given that the pre-labor uterine activity is manifested since the third trimester of pregnancy, and that BPRSA has been validated to measure the FCEC in labor, we hypothesized that BPRSA is a more reliable measure of FCEC than visual analysis and conventional measures such as cross-correlation, coherence, and cross-sample entropy. Additionally, by using BPRSA it is possible to detect FCEC even from the third trimester of pregnancy.

## 2. Materials and methods

### 2.1. Data collection

In this observational study, we acquired bioelectric transabdominal recordings of pregnant women at the third trimester of pregnancy (n = 37, from 32 to 39 gestational weeks). They attended the Maternal and Childhood Research Center (CIMIGen) in Mexico City, Mexico. The Ethics Committee of the Biological and Health Sciences Division (CBS) from Iztapalapa Campus of the Metropolitan Autonomous University (UAM) approved this research protocol (ref. CAEDCBS.01.2017). Written informed consent was obtained from each participant. We conducted this study following the Declaration of Helsinki and the institutional procedures of CIMIGen, UAM, and the Autonomous University of the State of Mexico (UAEMex).

Inclusion criteria were Mexican women in the third trimester of low-risk pregnancy, aged between 18 to 32 years old, and being residents of Mexico City or its metropolitan area. Additional inclusion criteria included pregnancy ending at term, normal body mass index (18.5 to 24.9 kg/m^2^), and normotensive participants. Women with multiple gestations, hypertension disorders in pregnancy, severe allergies, diabetes mellitus, autoimmune disease, renal dysfunction, maternal or fetal infection, and alcohol or drug abuse during pregnancy were excluded.

For data collection, we used a portable maternal-fetal monitor (Monica AN24^®^, Monica Healthcare, Nottingham, UK) [7]. The bioelectric transabdominal composite signals were recorded for 10 minutes using disposable electrodes (Ambu^®^ BlueSensor VL) in a bipolar configuration while women maintained a semi-Fowler’s position. The electrodes were positioned after cleaning the abdominal area with an alcohol swab and after carefully abrading the skin with sandpaper tape to reduce skin impedance.

### 2.2. Signals selection and preprocessing

We replicated the methodology conducted by Casati et al. [10], in which both transabdominal electrocardiogram and EHG were recorded at 900 Hz in the third trimester of pregnancy and stored for off-line analysis. The maternal uterine electrical activity was extracted from the slow wave of the EHG, i.e., its envelope, and the fetal heart rate (FHR) time series were extracted using the Monica DK software (version 1.9; Monica Healthcare). We discarded FHR and EHG time series if the loss of data was more than 50% in 10 minutes segments.

Such Monica’s software transforms the amplitude of the envelope of EHG into not calibrated numerical values, with the lowest possible value being 32 and the highest possible being 255 in arbitrary units (AU). To avoid the effects of fetal movements or maternal physical activity that can modify the envelope of the EHG as low amplitude oscillations, we only selected periods of true uterine contractions (identified by finding at least one uterine contraction in 10 minutes) showing a moderate or strong manifestation as reflected by EHG envelopes values between 50 to 101 AU [6].

The envelope of the EHG was obtained by a low-pass filtering the rectified fast-wave of the EHG (0.2 Hz to 1 Hz). The envelopes of the EHG and the FHR time series were averaged every two seconds to extract the profile of both signals. The FHR time series were reconditioned by a filtering approach and tested off-line in previous studies to exclude ectopic beats and artifacts [15]. Linear trends were removed from both FHR and EHG signals. Finally, we generated the corresponding BPRSA signals of each paired EHG-FHR signals as follows.

### 2.3. Generation of BPRSA signals

In each recording, the inter-relations between the envelope of EHG (as the trigger signal detected by increasing values of uterine activity) and the synchronous values of FHR (as the target signal) were analyzed using the BPRSA algorithm. According to the FCEC viewpoint, maternal uterine electrical activity triggers fetal heart rate changes (EHG→FHR). Thus, the modulations of the BPRSA target signal can be attributed to the periodicities of the trigger signal. If there are no interrelationships between the trigger (EHG) and target signal (FHR), the BPRSA target signal shows no periodic patterns (or these show a behavior closer to a flat line).

**Fig 1:**
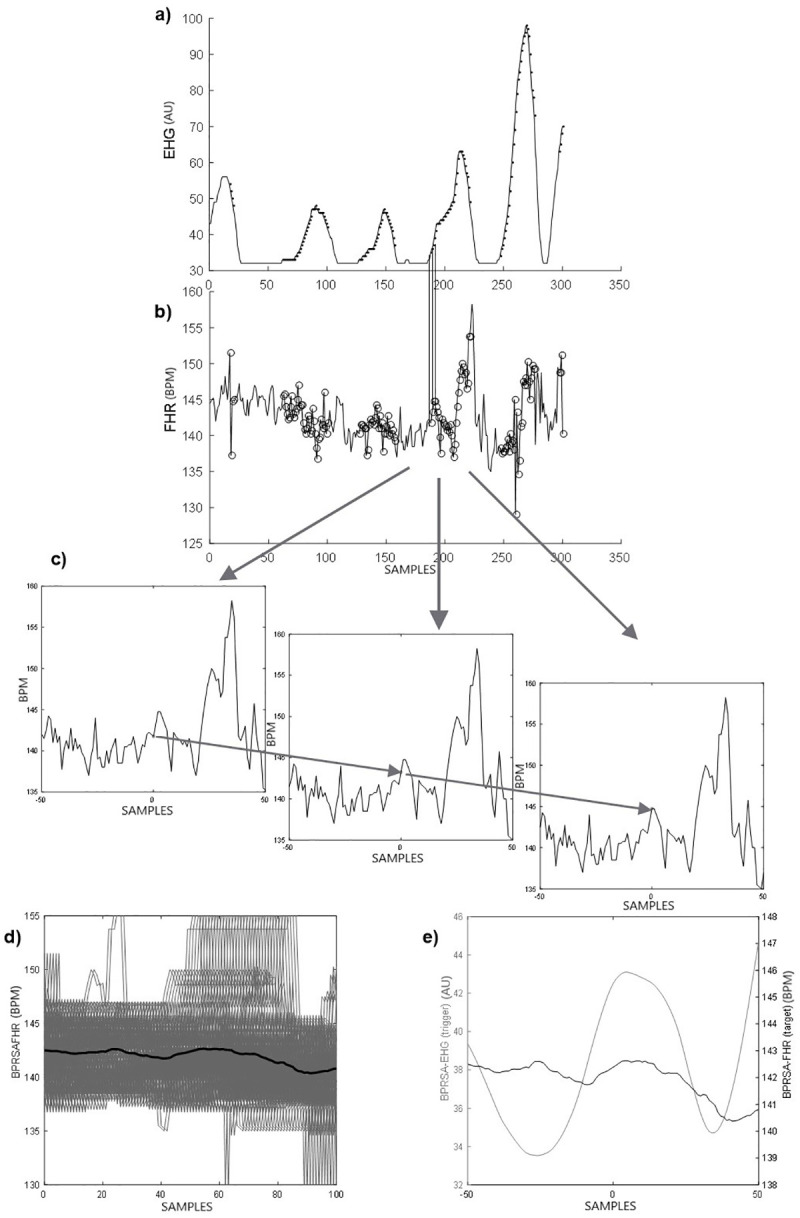
Description of the Bivariate Phase-rectified Signal Averaging (BPRSA) method: a) Raw uterine activity or electrohysterogram EHG (trigger signal): each data point depicted is the average of the raw signal obtained every 2 s. Each black dot represents an anchor point (EHG increases according to Eq 1). b) Raw fetal heart rate or FHR (target signal). The circles represent corresponding anchor points derived from the EHG signal; c) segments of fetal heart rate, centered around the anchor point (using time windows of 200 s, each data point (i) is the average of the FHR obtained every 2 s); d) Phase rectification at the anchor points and signal averaging; e) final BPRSA graph representing the BPRSA transformation of EHG and FHR.

The BPRSA algorithm consists of three steps [16]:

1. First, we identified the anchor points, A, in the envelope of the EHG signal (trigger) using the following equation:
  $$A=\frac{1}{T}\sum_{j=0}^{T-1}EHG\left(i+j\right)>\frac{1}{T}\sum_{j=1}^{T-1}EHG\left(i-j\right)$$ (1)
  We set a value of T = 18 proposed by Casati et al. to include the upper physiological frequency of uterine activity (0.01 Hz) and a window equal to 100 samples (200 seconds) was delimited around the anchor points [6].
2. The corresponding anchor points were identified using the time of occurrence of changes within the FHR signal (target) and are denoted as A*. The monovariate BPRSA signal contains all periodicities of the target signal; noise and nonstationary conditions were removed. Time frames of a certain length around each anchor A* are selected in the FHR signal.
3. All segments were aligned at the anchors leading to a phase-rectification of the segments. This procedure generates the BPRSA target signal and the BPRSA trigger signal. For further information, refer to Bauer [16]. In Fig 1, we summarize the steps of BPRSA analysis for the FHR and EHG signals.

The quantification of the FCEC by BPRSA and from the raw EHG and FHR signals using different approaches is described in the following section.

### 2.4. Quantification of FCEC in the BPRSA and raw signals

We quantified the coupling between the BPRSA trigger and target signals by the maximum coefficient of coherence (C_BPRSA_) [10], normalized cross-correlation coefficient (X-Corr_BPRSA_) [14,17], and cross-sample entropy (X-SampEn_BPRSA_) [18], which all have been applied in other studies to evaluate coupling between physiological signals.

For the maximum coefficient of coherence, we used the magnitude squared coherence method applied to a cross power spectral density analysis. The frequency content of the signals was calculated using a Welch-spectral analysis with a hamming window of 50 points and 50% overlap. We calculated the maximum coefficient of coherence (C_BPRSA_) between the BPRSA’s trigger and target signals (EHG and FHR), and we considered only significant coherence values higher than 0.50 [10]. The analysis of coherence was then repeated on the original or raw EHG and FHR signals for comparison, obtaining the C_RAW_ value.

In addition to that coherence, the similarity between the BPRSA’s trigger and target signals was evaluated by employing the normalized cross-correlation coefficient (X-Corr_BPRSA_). This value would be 1 if changes in the fetal heart rate depend entirely on changes in maternal uterine activity. For comparison, we also calculated the X-Corr_RAW_ from the raw signals. Analogous to the above interpretation for coherence, we also considered only significant cross-correlation values greater than 0.50.

The cross-sample entropy (X-SampEn) is a recently introduced parameter to measure the degree of asynchrony between two related time series. This parameter is negatively correlated according to the level of the nonlinear coupling between two time series [19]. In the X-SampEn analysis, larger values of cross-entropy suggest a weaker association and lower synchrony. We used an open-source software, which is available on PhysioNet for the computation of X-SampEn [20]. To our knowledge, no previous studies seem to have evaluated the FCEC using X-SampEn. We employed standard recommended ranges of embedding dimension m = 2 and a threshold value of r = 0.2 used in most of other related studies [21,22]. We also calculated the cross-sample entropy for BPRSA (X-SampEn_BPRSA_) and raw signals (X-SampEn_RAW_). All these calculations were done using Matlab^®^ software (the MathWorks, Inc. Natick, Massachusetts, USA).

### 2.5. Statistical analysis

In this study, we assessed significant differences between parameters evaluated from BPRSA signals (C_BPRSA_, X-corr_BPRSA_, X-SampEn_BPRSA_) and raw versions (C_RAW_, X-corr_RAW_, X-SampEn_RAW_) to compare the quantification of the FCEC. Normal distributions were tested by the Kolmogorov-Smirnov test. A two-tailed paired t-test or Wilcoxon matched-pairs signed-rank test were used to compare BPRSA and raw parameters. For all tests, results of p <0.05 were considered significant. The statistical analysis was carried out using the GraphPad Prism version 8.00 for Windows, (GraphPad Software, La Jolla California USA).

## 3. Results

The BPRSA analysis was applied to 37 FHR-EHG signals recorded in the late third trimester of pregnancy. The maternal/fetal general characteristics and the data of the pregnancy outcome of these participants are presented in Table 1. No major complications occurred in newborns as indicated by weights and Apgar scores at 1 minute and 5 minutes. (The Apgar test is used to quickly identify the health of newborns according to the assessment of heart rate, respiration, color, muscle tone, and reflexes).

**Table 1:** Maternal and newborn clinical characteristics of the studied cases (n = 37).

Description	
Maternal age (years)	25.0, [20.0, 29.5]
Gestational age (weeks)	37, [36, 38]
Maternal weight (kg)	66.5, [64.1, 74.4]
BMI (kg/m^2^)	27.5, [24.7, 31.1]
Waist circumference (cm)	103.0, [96.5, 108.7]
Hip circumference (cm)	105.0, [100.0, 110.5]
Caesarean section (n, %)	15 (40)
Fetal gestational age-parturition (weeks)	39.55, [39.0, 40.3]
Apgar	
1 Minute	8.0, [8.0, 8.1]
5 Minutes	9.0, [9.0, 9.1]
Birth Weight (kg)	3.28, [2.93, 3.56]
Head Circumference (cm)	33.5, [32.5, 35.0]
Length (cm)	50.0, [49.0, 50.5]
Gender (n, % male)	13 (35)

Values expressed as median (interquartile range) unless otherwise indicated

**Fig 2:**
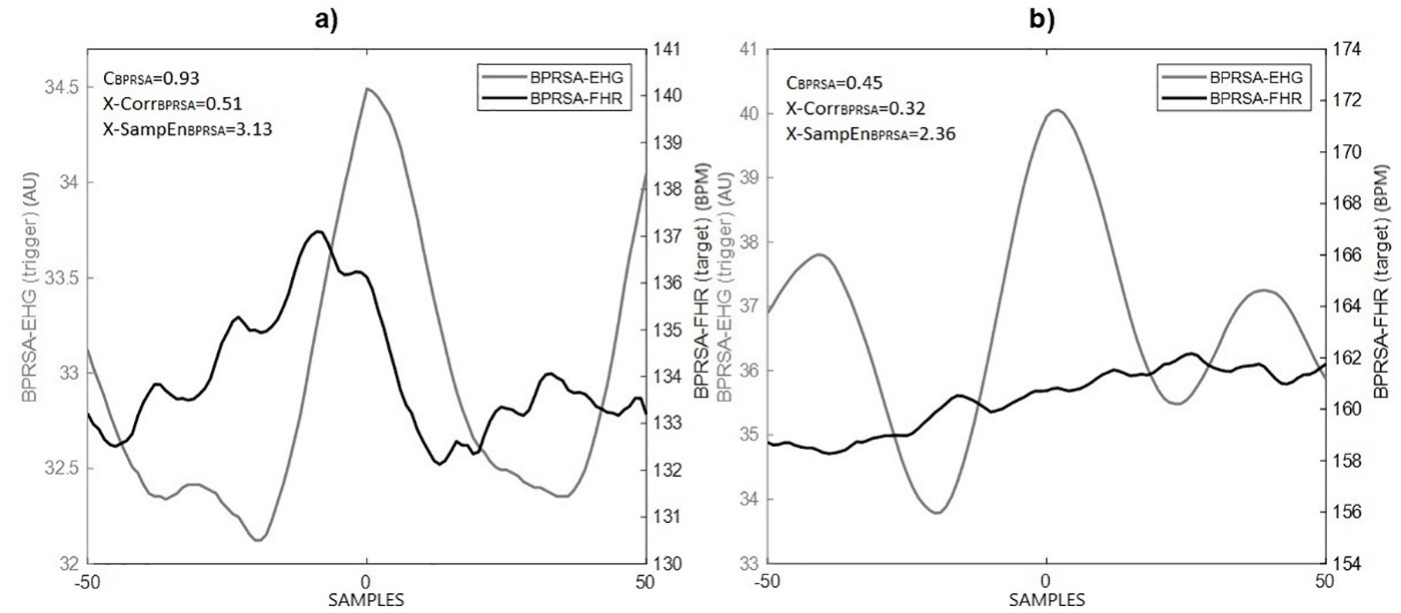
A representative example of Bivariate Phase-rectified Signal Averaging (BPRSA): The grey line depicts the BPRSA transformation of the electrohysterogram (EHG) signal. The black line depicts the BPRSA transformation of the fetal heart rate (FHR) changes in correspondence with the EHG. The time axis, centered around time = 0 (aligned anchors), permits to observe the oscillation of the signals before and after the triggering event (time windows of 200 seconds, each data point *(i)* is the average of BPRSA obtained every 2 seconds). Panel a: shows a manifestation of cardio-electrohysterographic coupling (black line oscillation); panel b: shows the absence or low coupling (flat-like line). The values of coherence (C_BPRSA_) and cross-correlation (X-Corr_BPRSA_) are higher in the presence of fetal cardio-electrohysterographic coupling.

Following a visual interpretation of the BPRSA graphs, the presence of a central oscillation in the BPRSA signal indicates coupling (FCEC) between the uterine electrical activity and FHR (Fig 2a). Otherwise, the absence or low coupling is exhibited as a flat-like line (Fig 2b). Maximum coherence coefficient or normalized cross-correlation values greater than 0.50 also indicate the presence of coupling between the two signals.

According to the values of C_RAW_, the FCEC was detected in 48% cases (18/37) and using C_BPRSA_ in 92% cases (34/37). In line with these results, the X-Corr_BPRSA_ indicated FCEC in 82% cases (30/37) compared to 30% cases (11/37) using X-Corr_RAW_. A standard value for evaluating the presence of coupling using cross-sample entropy has not been defined [23]; however, 92% cases (34/37) exhibited lower X-SampEn_BPRSA_ values than those obtained with the raw parameter.

**Fig 3:**
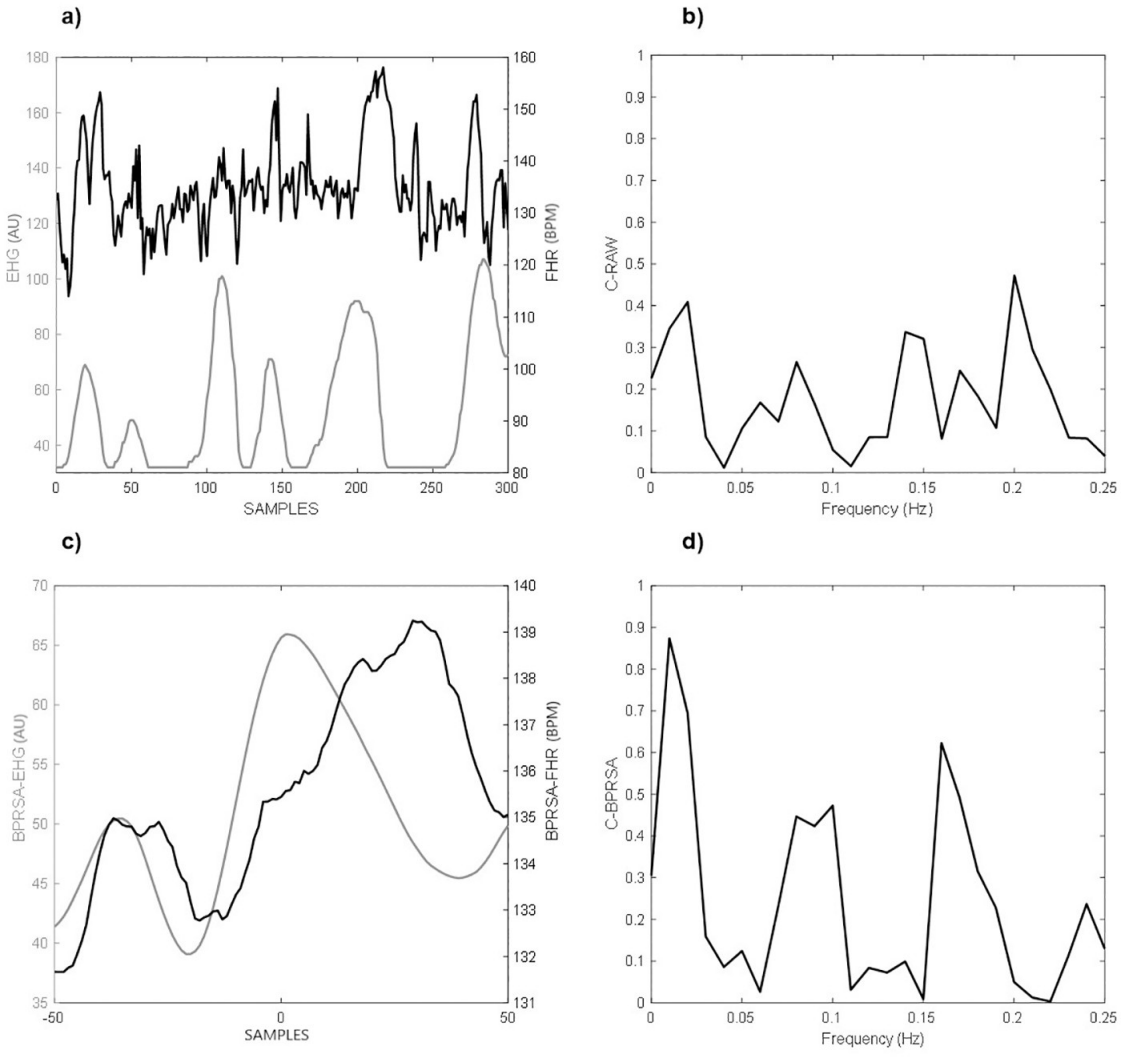
A representative comparison of Bivariate Phase-rectified signal averaging (BPRSA) as applied to raw signals: a) 10 minutes recording of electrohysterogram (EHG, grey line) and fetal heart rate (FHR, black line) without any signal loss; b) maximum coefficient of coherence for the raw signals (CRAW); c) the BPRSA graph shows coupled periodicities; d) maximum coefficient of coherence for BPRSA signals (CBPRSA), values greater than 0.5 are considered as meaningful.

To provide a representative example of our analysis, in Fig 3a, we show an example of the FHR and EHG raw signals. Visually, the FHR seems to increase in correspondence with higher uterine activity. However, the values of C_RAW_ revealed no meaningful coupling between signals (values lower than 0.50 in Fig 3b). In contrast, when we generated the BPRSA graphs of the FHR and EHG signals, a central oscillation does indicate the presence of coupling between the uterine electrical activity and FHR (Fig 3c). This coupling was confirmed by values of C_BPRSA_ greater than 0.50 (Fig 3d).

**Fig 4:**
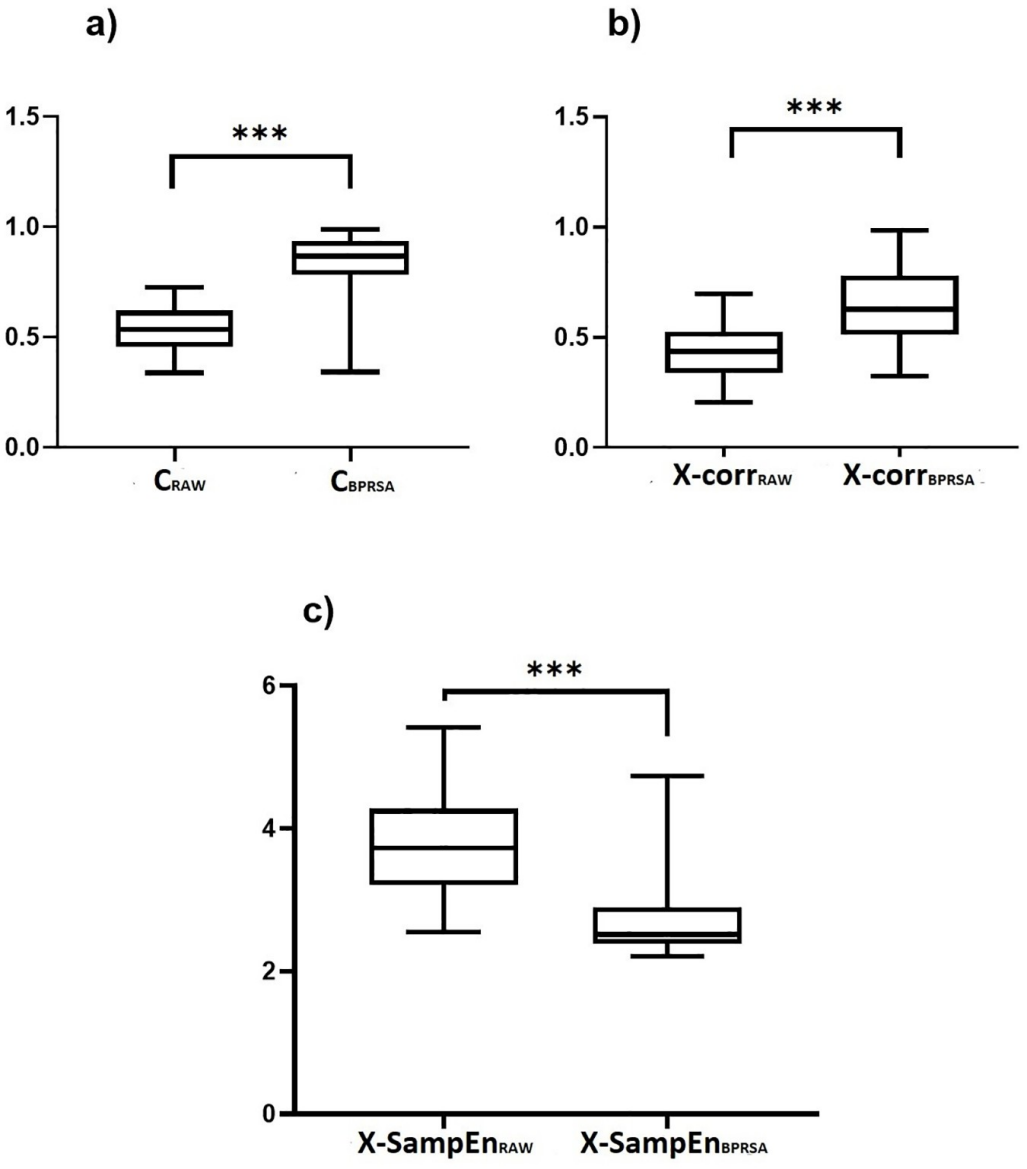
Comparison between the raw parameters of fetal heart rate (FHR) and electrohysterogram (EHG) coupling at the third trimester of pregnancy provided either by the maximum coefficient of coherence (C_RAW_), the normalized cross-correlation coefficient (X-corrr_RAW_) and cross-sample entropy (X-SampEn_RAW_) or by the Bivariate Phase-Rectified Signal Averaging versions (BPRSA) of the maximum coefficient of coherence (C_BPRSA_), the normalized cross-correlation coefficient (X-corrr_BPRSA_) and the cross-sample entropy (X-SampEn_BPRSA_): a) C_RAW_ vs. C_BPRSA_ (0.53±0.10 vs. 0.82±0.15); b) X-Corr_RAW_ vs. X-Corr_BPRSA_ (0.42±0.12 vs. 0.63± 0.17) and c): X-SampEn_RAW_ vs. X-SampEn_BPRSA_ (3.70±0.65 vs. 2.68±0.48), respectively. *p<0.0001 according to Wilcoxon matched-pairs signed-rank test.

Overall, we found significant differences between the raw parameters obtained from original and BPRSA signals. For the coherence and cross-correlation parameters, the values were significantly higher for the parameter BPRSA versions in comparison to the raw versions: C_RAW_ vs. C_BPRSA_ (0.53±0.10 vs. 0.82±0.15, p<0.0001, Fig 4a), X-Corr_RAW_ vs. X-Corr_BPRSA_ (0.42±0.12 vs. 0.63± 0.17, p<0.0001, Fig 4b), respectively. Finally, the cross-sample entropy parameter exhibited significantly lower values for the BPRSA version in comparison to the raw version: X-SampEn_RAW_ vs. X-SampEn_BPRSA_ (3.70±0.65 vs. 2.68±0.48, p<0.0001, Fig 4c), respectively.

## 4. Discussion

There are few studies regarding the interrelationship between uterine activity and FHR in the antenatal period [6]. We found evidence that the antenatal uterine activity influences the FHR in low-risk healthy women in the third trimester of pregnancy. Our results indicate that the fetal cardio-electrohysterographic coupling (FCEC) is manifested at the third trimester of pregnancy in healthy women few weeks before labor.

Additionally, other novel findings show that the manifestation of contractions before labor, which appears to occur in all species, can also affect the fetal behavioral activity [24]. In sheep, the increasing frequency of these uterine contractions above the normal rate has maturational effects on the neural and cardiovascular function [24]. The antenatal uterine activity produces stress to the fetus and an increased need for blood flow to the placenta to provide fetal oxygenation [25]. Thus, the FCEC may play a key role in the healthy physiological development of the fetus in the third trimester of pregnancy.

Yet, contrary to our results, a study found that antenatal contractions have little or no effect on both fetal hemodynamics and oxygenation in the healthy near term human fetus [26]. This discrepancy is likely caused by the use of different data acquisition methods: electrohysterography vs. tocodynamometry or Doppler ultrasound vs. fetal electrocardiography. Presumably, the small decrease in fetal oxygenation provokes a small reduction in the cerebral vascular resistance, which is not detectable with Doppler ultrasound [26].

Our results are in line with Casati et al. [10], who showed that visually the BPRSA analysis revealed the FCEC (Fig 2a). This coupling was confirmed here by the mathematical computation of the maximum coefficient of coherence and the normalized cross-correlation coefficient. In general, the BPRSA analysis provided higher estimates than those obtained using the raw signal analysis to detect and quantify the FCEC. Analogous to Casati et al., the quantification of FCEC using BPRSA distinguished coupling in participants for whom the traditional raw analysis failed to identify any coupling (Fig 3). This finding may be related to the consideration that FHR and EHG in the third trimester of pregnancy involve a high degree of nonstationarities and noise [16].

We also introduced here the cross-sample entropy (X-SampEn) as an additional parameter for the assessment of coupling between FHR and EHG. We found that the quantification of the BPRSA graphs by X-SampEn produces lower values of this parameter in comparison to those obtained by the raw analysis (Fig 4c). Notably, larger values of X-SampEn are associated with decoupling, while lower values represent a higher coupling [18]. Thus, the evaluation of X-SampEn in BPRSA graphs seems to confirm the presence of FCEC in the third trimester of pregnancy in coincidence with the findings by the coherence (Fig 4a) and cross-correlation analysis (Fig 4b).

Although recruiting high-risk pregnant patients was not in the scope of this study, the estimation of FCEC could also play a role to identify and get further insights into this condition. Interestingly, other studies have already assessed the FCEC in high-risk pregnancies of small for gestational age fetuses using other mathematical approximations. For example, Chen et al. indicated that the assessment of the interaction between FHR and EHG might provide new information for early detection and a comprehensive interpretation in prenatal diagnosis, becoming helpful for improving the screening of intrauterine growth restriction [11]. Another novel clinical application has proposed that the quantification of FCEC could be used to derive robust features for the classification of the dynamics of vaginal vs. cesarean childbirth [27].

We consider that the clinical use of the BPRSA as a reliable technique to measure the influence of EHG on FHR in the antenatal period is promising. In fact, the assessment of the fetal response to uterine activity by BPRSA in the antenatal period as an early marker of fetal distress in conjunction with the recognition of FHR patterns could be associated with the state of fetal oxygenation, which is an important area of clinical interest in obstetrics [28]. However, the clinical interpretation of different patterns of coupling (or even the lack of coupling) between EHG and FHR assessed by BPRSA still needs to be elucidated. Likewise, further mathematical approximations should be tested for the quantification of FCEC of both signals in high-risk pregnancy patients.

In the present report, we did not acquire simultaneous ultrasound images to visualize fetal body or eye movements, which are useful to discriminate among fetal behavioral states [29,30]. Thus, such different fetal behavioral states could have introduced significant effects on our FCEC estimates.

## 5. Conclusions

We found that fetal cardio-electrohysterographic coupling (FCEC) is manifested in the third trimester of pregnancy in healthy women, showing that the fetal heart rate is influenced by the pre-labor uterine activity as it also occurs during labor. The persistence of this physiological mechanism before the onset of labor may play a key role in the healthy development of a graded physiological response of the fetus at the antenatal period. The use of the BPRSA analysis confirmed that it is reliable when applied to FHR and EHG transabdominal data and for the quantification of the FCEC during the late third trimester of pregnancy.
